# Occupationally related bilateral calcific tendonitis of Flexor carpi ulnaris: case report

**DOI:** 10.1186/1749-799X-4-33

**Published:** 2009-08-23

**Authors:** Mark Edmondson, Andrew Skyrme

**Affiliations:** 1 Eastbourne District General Hospital, Kings Drive, Eastbourne, BN21 2UD, UK

## Abstract

We present a case of bilateral calcific tendonitis of the Flexor Carpi Ulnaris attributable to repetitive wrist action which was occupationally related. This was treated conservatively with avoidance of aggravating movement, resting splints and anti inflammatory medication when acute flare ups occurred. Since avoidance of repetitive strain on the wrists he has had no further flare ups in over 2 years. This is the only case of bilateral calcific tendonitis of Flexor Carpi Ulnaris that has been reported in the literature, further more it is the only one which has been attributed to occupation and settled following a change of career.

## Introduction

Calcium deposits may occur in virtually any tendon or ligament although they are more common in the rotator cuff of the shoulder. Calcific deposits tend to arise in tendons secondary to chronic inflammation. Chronic inflammation can arise from repetitive strain injuries resulting from repeated stress to the body's soft tissues. They often occur in patients who perform repetitive movements either in their jobs or in extracurricular activities. However it is not uncommon to find calcific deposits within the tendons of patients who cannot recall any provocative factors. We report a case of bilateral calcific tendonitis of Flexor Carpi Ulnaris tendons at the wrist which correlates with occupationally related repetitive strain.

## Case report

**Figure 1:**
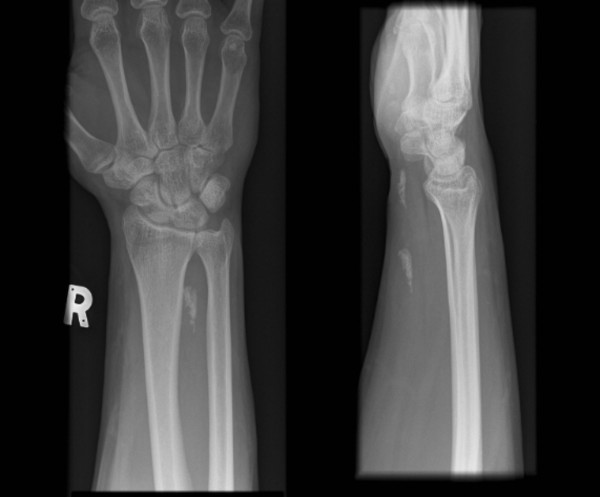
**Radiographs of Right wrist demonstrating Calcific tendonitis of Flexor Carpi Ulnaris**.

A 42 year old male Hospital porter presented to our outpatient clinic with bilateral wrist pain. The pain was localised to the volar and ulna aspect of both wrists with specific tenderness along the course of the Flexor Carpi Ulnaris tendon proximal to the pisiform. There was palpable nodularity throughout these tendons. The pain was aggravated by flexion and ulna deviation of the wrist during examination in clinic. His right wrist was worse than his left, and he attributed this to his work as a porter which involved turning patients' beds to the right and left which significantly strained his forearm flexors (and specifically the ulna sided ones to flex the wrist). Xray examination revealed bilateral calcific tendonitis of Flexor Carpi Ulnaris (Figure 1).

He does not suffer from hypercalcaemia, have any metabolic abnormalities and does not suffer from CREST syndrome (Calcinosis Raynauds Esophagitis Scleroderma Telangiectasia).

He was managed conservatively with avoidance of provocative movement, resting splints and NSAIDS for painful exacerbations. The patient infact changed his career to work in the mortuary rather than as a porter and this aided in avoiding exacerbation of his pain.

On review, 2 years since original presentation, he has had no further flare ups since changing careers.

It is also noteworthy that the calcific deposits were still present on radiographic examination of his right wrist at this stage (Figure 1) despite resolution of symptoms, but not in his left.

## Discussion

A calcium deposit at the insertion of Flexor Carpi Ulnaris following trauma was first described by Cohen in 1924 [1]. Calcareous tendonitis in the metacarpophalangeal joint region was reported by Cooper in 1942 [2], presenting with severe local tenderness, swelling and erythema. Carrol et al described the commonest site for calcific deposits along the Flexor Carpi Ulnaris to be near the pisiform [3], this has further been described as calcification within the pisiform bursa [4]. Yelton and Dickey described 97 cases of calcification around the hand and wrist 2/3 s of which were in the metacarpophalangeal region [5], they found that injection of local anaesthetic in the acute stages gave lasting relief and postulated that the physical act of needling the deposit produced this result. More recently Moyer et al described 12 cases of calcific tendonitis of the hand and wrist which was deemed to be idiopathic as no initiating trauma or repetitive strain could be identified [6]. Pathogenesis of calcific tendonitis remains unclear, although tendon fibre damage, dystrophic calcification, and hypoxia have been implicated [7].

Calcific tendonitis can present with severe pain and tenderness along the tendon coupled with intense erythema. Presentations like this can often raise the suspicion of infection, it is worth bearing in mind the possibility of calcific tendonitis in such cases. Review of the literature revealed no cases of either bilateral Flexor Carpi Ulnaris Calcific tendonitis or occupationally related calcific tendonitis of the Flexor Carpi Ulnaris. Our case is unusual in that it is bilateral and related to repetitive strain.

## Consent

Written informed consent was obtained from the patient for publication of this case report and any accompanying images. A copy of the written consent is available for review by the Editor-in-Chief of this journal.

## Competing interests

The authors declare that they have no competing interests.

## Authors' contributions

Both authors were involved with the assessment and subsequent follow up of this patient. Both authors read and approved the manuscript.

## Author information

Mark Edmondson is a Trauma and Orthopaedics Registrar and Andrew Skyrme is a Trauma and Orthopaedic Consulant at the Eastbourne District General Hospital, Kings Drive, Eastbourne, BN21 2UD, UK.
